# Genome-wide analysis of expression QTL (eQTL) and allele-specific expression (ASE) in pig muscle identifies candidate genes for meat quality traits

**DOI:** 10.1186/s12711-020-00579-x

**Published:** 2020-10-09

**Authors:** Yan Liu, Xiaolei Liu, Zhiwei Zheng, Tingting Ma, Ying Liu, Huan Long, Huijun Cheng, Ming Fang, Jing Gong, Xinyun Li, Shuhong Zhao, Xuewen Xu

**Affiliations:** 1 Key Laboratory of Agricultural Animal Genetics, Breeding and Reproduction, Ministry of Education & College of Animal Science and Technology, Huazhong Agricultural University, Wuhan, 430070 China; 2 The Cooperative Innovation Center for Sustainable Pig Production, Wuhan, 430070 China; 3 Key Lab of Swine Genetics and Breeding of Ministry of Agriculture and Rural Affairs, Wuhan, 430070 China; 4 Colleges of Informatics, Huazhong Agricultural University, Wuhan, 430070 China; 5 Key Laboratory of Healthy Mariculture for the East China Sea, Ministry of Agriculture and Rural Affairs, Fisheries College, Jimei University, Xiamen, 361021 China

## Abstract

**Background:**

Genetic analysis of gene expression level is a promising approach for characterizing candidate genes that are involved in complex economic traits such as meat quality. In the present study, we conducted expression quantitative trait loci (eQTL) and allele-specific expression (ASE) analyses based on RNA-sequencing (RNAseq) data from the *longissimus* muscle of 189 Duroc × Luchuan crossed pigs in order to identify some candidate genes for meat quality traits.

**Results:**

Using a genome-wide association study based on a mixed linear model, we identified 7192 cis-eQTL corresponding to 2098 cis-genes (p ≤ 1.33e-3, FDR ≤ 0.05) and 6400 trans-eQTL corresponding to 863 trans-genes (p ≤ 1.13e-6, FDR ≤ 0.05). ASE analysis using RNAseq SNPs identified 9815 significant ASE-SNPs in 2253 unique genes. Integrative analysis between the cis-eQTL and ASE target genes identified 540 common genes, including 33 genes with expression levels that were correlated with at least one meat quality trait. Among these 540 common genes, 63 have been reported previously as candidate genes for meat quality traits, such as *PHKG1* (q-value = 1.67e-6 for the leading SNP in the cis-eQTL analysis), *NUDT7* (q-value = 5.67e-13), *FADS2* (q-value = 8.44e-5), and *DGAT2* (q-value = 1.24e-3).

**Conclusions:**

The present study confirmed several previously published candidate genes and identified some novel candidate genes for meat quality traits via eQTL and ASE analyses, which will be useful to prioritize candidate genes in further studies.

## Background

In the past 10 years, genome-wide association studies (GWAS) have dramatically accelerated the forward genetic dissection of complex traits in various species. For example, according to the release (as of 2019 September 30) of the animal quantitative trait loci (QTL) database (https://www.animalgenome.org/), 16,085 QTL or associations for pork quality and carcass traits have been reported. However, previous reported GWAS results revealed that most of the leading single nucleotide polymorphisms (SNPs) for complex traits were located in noncoding regions [1], which suggests that variants located in regulatory elements contribute to phenotypic variation by regulating gene expression. Thus, prioritizing the functional genes and related causal variants that underlie QTL, especially for noncoding regions, is the primary challenge in the post-GWAS era. Integration analysis of molecular phenotypes is anticipated to be one of the most promising approaches to solve this challenge [2–4].

Variants that regulate gene expression level, i.e. expression QTL (eQTL), are well known to contribute significantly to phenotypic variation [5]. Cis-eQTL analysis can highlight the functional candidate genes in the characterized QTL region [4, 6, 7]. Trans-eQTL and coexpression analysis can uncover trans-acting factor-mediated networks that control phenotypic variation [8, 9]. Thus, eQTL analysis bridges the gap between genotype and phenotype [10]. Recently, integration analyses of GWAS and eQTL have revealed clear advantages for prioritizing causal genes for human diseases [11–15], and statistical methods for such analyses have been developed [16–18].

In pigs, genome-wide eQTL analysis of the *longissimus* muscle has been conducted based on cDNA microarrays in four populations [19–23] and has deepened our understanding of the genetic variability of gene expression in muscle and provided novel candidate genes for meat quality-related traits. With the rapid decline of sequencing costs, RNA sequencing (RNAseq) is quickly replacing cDNA microarrays to achieve high-throughput assessment of gene expression levels and has recently been used for eQTL analysis related to porcine disease resistance [24] and in blood [25], skeletal muscle [26], and testis [27]. RNAseq also provides sequence information that enables the measurement of allele-specific expression (ASE), which could provide further confirmation of the results of cis-eQTL mapping [28]. An integrated strategy that combines eQTL and ASE analyses has been reported in pigs [25] and cattle [29].

In the present study, we characterized eQTL in porcine skeletal muscle by combining genome-wide eQTL and ASE analyses based on RNAseq data from 189 animals, in order to better understand the genetic regulation of gene expression in this tissue and to identify candidate genes that affect meat quality traits.

## Methods

### Animal sampling and phenotyping

The experimental population was described in our previous study [30]. Briefly, 425 animals (hereafter referred to as DL) produced by crossing eight Duroc boars with 158 Luchuan sows were fed following the same protocol and slaughtered by a standardized procedure at the age of 210 ± 6 days. The *longissimus dorsi* muscle at the junction of the thoracolumbar was removed and immediately frozen in liquid nitrogen for RNA extraction, and the spleen was sampled for DNA extraction. The current study was approved by the Scientific Ethics Committee of Huazhong Agricultural University (the approval number is HZAUSW-2016-010), Wuhan, China.

We measured the following meat quality traits of the *longissimus dorsi* muscle: meat color parameters L*, A*, B*, C and H, pH value at 45 min (pH 45 min) and 24 h (pH 24 h) post-slaughter, and drip loss. All procedures followed the Agricultural Industry Standards “Determination of Livestock and Poultry Meat quality” (NY/T 1333-2007) of the People’s Republic of China.

### DNA extraction, genotyping, and quality control (QC)

Genomic DNA was isolated from the spleen of 189 DL pigs (a randomly selected subset of the DL population mentioned above) by the standard method of phenol–chloroform extraction and was quantified with a NanoDrop-2000 spectrophotometer (Thermo Scientific). These individuals were then genotyped using the Illumina porcine 50 K + SNP iSelect™ BeadChip at Neogen Bio-Scientific Technology (Shanghai) Co., Ltd.) according to the manufacturer’s protocol. The genotyping results of all individuals were merged with the “merge” function of plink (http://pngu.mgh.harvard.edu/purcell/plink/) [31]. Quality control (QC) was done using Plink, with the following parameters: a threshold for minor allele frequency of 1% (–maf 0.01), a missing genotype call rate per SNP lower than 10% (–geno 0.1) and a missing genotype call rate per sample lower than 15% (–mind 15). Then, the missing genotypes were imputed with Beagle v4.1 by setting default parameters [32]. To avoid an extreme distribution of genotypes, we counted the number of individuals in each genotype by SNP and then calculated the median of the numbers of each genotype at each SNP. Only the SNPs on the autosomes and the X chromosome with a median number larger than 10% of the total number (≥ 19) were kept. Finally, 36,045 SNPs passed all criteria and were retained for further analysis.

### RNA extraction, sequencing, and quality control

Total RNA was extracted from the *longissimus dorsi* muscle of all 189 individuals with the Trizol reagent according to the product manual. RNA purity and integrity were assessed using an Agilent 2100 bioanalyzer (Agilent Technologies) and quantified with a NanoDrop-2000 spectrophotometer (Thermo Scientific). For each sample, one µg of total RNA was used for the construction of a 2 × 150 bp paired-end mRNA sequencing library with the NEBNext^®^ UltraTM RNA Library Prep kit for Illumina^®^ (NEB, USA). RNA sequencing reactions were conducted on the Illumina HiSeq 4000 platform. The RNAseq data produced were cleaned through the following QC steps: (1) remove adaptor; (2) discard reads containing more than 5% N; and (3) remove low-quality reads (the percentage of bases with quality score < 20).

### eQTL mapping

The cleaned data were aligned to the reference genome assembly Sus_scrofa 11.1 (Ensembl Release 90), using Hisat2 v2.0.5 with a transcript annotation index [33]. The expression levels of genes annotated in the reference genome were quantified with StringTie v1.3.3 [34]. A TPM (transcripts per kilobase per million mapped reads, TPM) value of 0.01 was considered as a minimum threshold for a gene to be regarded as expressed, and only those genes that were expressed in 90% of the samples were kept for eQTL analysis. Prior to eQTL analysis, normalization was performed for each gene separately based on the rank of TPM values across samples.

In this study, eQTL analysis was carried out with MatrixEQTL [35] based on the following fixed linear model,

$${\text{Y}}\,\text{ = }\;\upbeta * {\text{SNP}}\;\text{ + }\;{\text{PC}}\;\text{ + }\;{\text{G}}\;\text{ + }\;{\text{Ba}}\;\text{ + }\;{\text{Bo}}\;\text{ + }\;{\text{A}}\,\text{ + }\;{\text{e}},$$

where Y is the normalized expression level of the target gene, β is the SNP allele substitution effect, SNP is the marker genotype covariate, coded 0 (for homozygotes for the reference allele), 1, and 2 [35], PC are the top five principal components for correcting for population stratification, which were calculated using the prcomp function in R based on marker genotypes, ${\text{G}}$ is gender, ${\text{Ba}}$ is slaughter batch, ${\text{Bo}}$ is the effect of boar, ${\text{A}}$ is a covariate of age, and ${\text{e}}$ is a random residual. The cis-eQTL mapping window was defined from 1 megabase (Mb) upstream of the transcription start site to 1 Mb downstream of the gene end; all other SNP-gene pairs were defined as trans-associated. For both cis- and trans-eQTL, we set the adjusted p value (q value) via the method of false discovery rate (FDR) with a significance threshold at q = 0.05. Heritability of the expression level for each gene was estimated with HIBLUP (https://github.com/xiaolei-lab/hiblup) using a mixed linear animal model with sex, age, slaughter batch, and boar as fixed effect terms and the genomic relationship matrix that derived from all available DNA chip SNPs as a random effect term. Student’s t test was used to compare differences in heritability between groups of genes.

### Allele-specific expression analysis based on RNAseq SNPs

Variants were called with GATK from the aligned data according to the best-practice pipeline (https://gatkforums.broadinstitute.org/gatk/discussion/3891/calling-variants-in-rnaseq). QC was performed for all obtained RNAseq variants using Plink [31] with the following parameters: threshold for the minor allele frequency of 1% (–maf 0.01) and a missing genotype call rate lower than 10% (–geno 0.1). Then the missing genotypes were imputed with Beagle v4.1 with default parameters [32]. Subsequently, we removed Insertion/Deletion variants and only kept the SNPs that were on 18 autosomes and the X chromosome for further analysis. By overlap analysis, we identified all detected SNPs that were common to the DNA chip and RNAseq. With the genotype of the DNA chip as reference, the genotyping reliability of RNAseq SNPs was evaluated using the common SNPs with consistency and precision rate of heterozygotes (the number of true positive heterozygotes divided by the total number of true and false positive heterozygotes).

To avoid allelic mapping bias, we built a N-masking genome based on RNAseq SNPs with the maskFastaFromBed script in bedtools v2.26.0 [36]. The clean RNAseq data were realigned to the N-masking genome using Hisat2 v2.0.5 with a transcript annotation index [33]. Then, allele-specific reads were calculated using the GATK ASEReadCounter tool [37]. For each sample, the informative SNPs were singled out using the following parameters: at least 10 total reads and 3 allele specific reads, and a percentage of specific reads for each allele higher than 1% of the total mapped reads [38]. Then, allele specific expression was evaluated for each informative SNP using a binomial exact test in R, with the FDR level set to 5% across SNPs per sample. Several additional filtering conditions for ASE SNPs were set at the population level: at least 30 heterozygotes and at least 10 heterozygotes that displayed ASE. Finally, the ASE SNPs were annotated with snpEff [39].

### Correlation analysis between gene expression and meat quality traits

The phenotypic and log_2_-transformed gene expression data were corrected using a linear model, with sex, slaughter batch, and boar as fixed effects and age as a covariate. Pearson correlation coefficients were calculated between the residuals of log_2_-transformed expression levels of each gene and corrected phenotypic values of seven traits. For each trait, the q value was calculated using the R function p.adjust via the FDR method, with q ≤ 0.05 as the significance threshold for the correlations.

### Collection of candidate genes from published references

To highlight potential candidate genes for the trait-associated genes that were identified by the eQTL and ASE analyses, we collected all published references with the key words “pig” and “GWAS” in the database of PubMed (https://www.ncbi.nlm.nih.gov/pubmed). The obtained publications were inspected for content to identify publications that were related to pig genetics and names of candidate genes were collected. The Ensembl Gene ID of the candidate genes were obtained by the Biomart tool, and overlap analyses were conducted using their Ensembl Gene ID to identify candidate genes from cis-eQTL- or ASE-associated genes.

## Results

### eQTL identified by GWAS

In total, RNA sequencing of the *longissimus dorsi* muscle of 189 DL pigs yielded 9.23 billion clean reads with a length of 150 bp, a total size of 1385.20 gigabases (Gb), and an average sequencing size of 7.33 Gb, ranging from 5.66 to 10.78 Gb (see Additional file 1: Table S1). The expression levels of all reference genes were estimated for the 189 individuals with StringTie and 13,450 genes were kept for further analysis after QC. As for SNP genotyping, 188 individuals had a genotype call rate higher than 94% and one individual had a genotype call rate of 85.37%. After QC, 36,045 SNPs for 189 individuals were retained for further analysis.

**Fig. 1:**
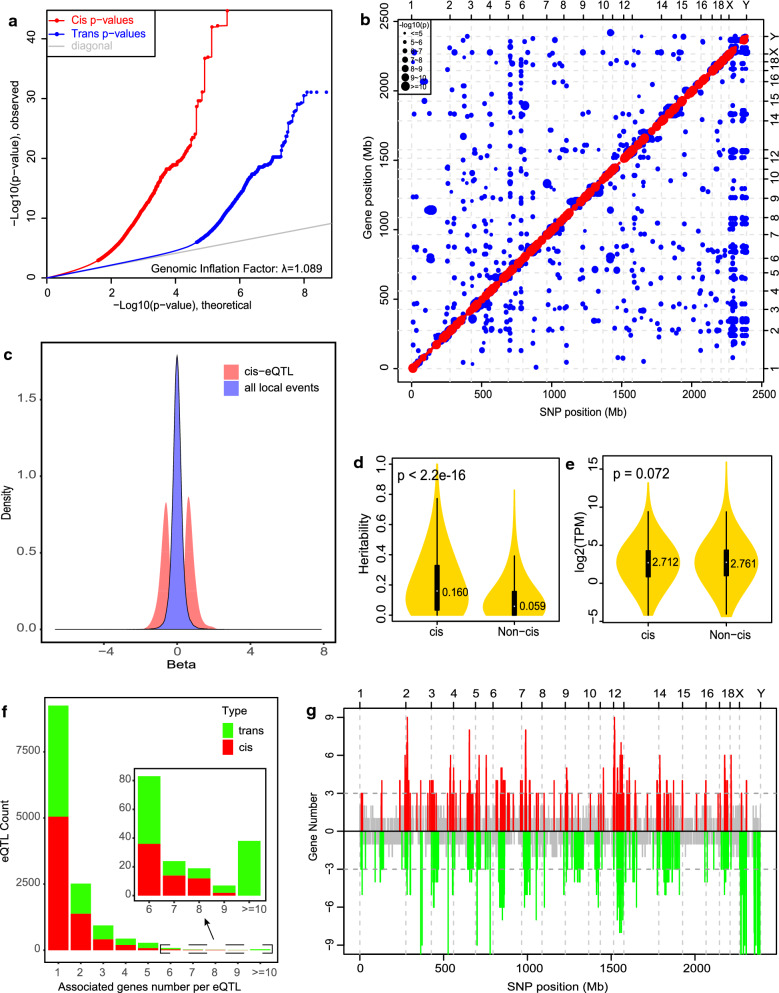
Genome-wide eQTL analysis. **a** QQ-plot of -Log10(*p* value) of eQTL analysis using MatrixEQTL. “Cis p values” represent the statistical p values for cis-eQTL and “Trans p values” represent the statistical p values for trans-eQTL. **b** Scatter plot of all characterized eQTL. Each dot represents a SNP-gene pair, with the vertical direction linking to the SNP and the horizontal direction linking to the gene. The red and blue dots represent cis-eQTL and trans-eQTL respectively. The size of the dot indicates the degree of significance. **c** Density distribution of the cis-eQTL effects. “all local events” represents all the SNP-gene pairs for which the distance between SNP and gene is less than 1 Mb. **d** Comparison of the heritability of the expression levels of cis-eQTL associated genes and that of non-cis-eQTL genes. The white dots represent the median values. The p value indicates the difference between the two groups, which was calculated using a two-tailed t-test. **e** Comparison of the expression levels of cis-eQTL-associated genes and non-cis-eQTL genes. The white dots represent the median values. **f** Analysis of eQTL pleiotropy. The X-axis of the histogram represents different groups that were classified according to the associated gene numbers per eQTL, and the Y-axis represents the eQTL count for each group. cis-eQTL and trans-eQTL are distinguished by different colors. **g** Distribution of eQTL hotspot. The X-axis represents the chromosome distribution of eQTL, the Y-axis indicates the count of genes associated with each eQTL. The upper part displays the cis-eQTL hotspot distribution, and if the count of associated genes is greater than 3, it is shown in red. The lower part in displays the trans-eQTL hotspot distribution, and if the count of associated genes is greater than 3, it is shown in green

The eQTL analysis assessed associations between SNPs and gene expression levels for 401,736 cis- and 484,403,514 trans-SNP-gene pairs (Fig. 1a). The calculated genomic inflation factor was 1.089, which indicated that the model controlled potential population stratification well. The QQplot in Fig. 1a showed that the distribution of the observed p values of cis-eQTL displayed earlier departure from the diagonal than that of trans-eQTL, which indicated that cis-eQTL were easier to detect than trans-eQTL. In total, we identified 10,693 significant cis-SNP-gene associations (p ≤ 1.33e-3, q-value ≤ 0.05) (red dots on the diagonal line of Fig. 1b), corresponding to 7192 cis-acting SNPs (cis-SNPs) and 2098 cis-eQTL-associated genes (cis-genes) (see Additional file 2: Table S2). We also characterized 10,961 trans-SNP-gene associations (p ≤ 1.13e-6, q-value ≤ 0.05) (blue dots in Fig. 1b), corresponding to 6400 trans-acting SNPs (trans-SNPs) and 863 trans-eQTL-associated genes (trans-genes) (see Additional file 3: Table S3).

Intersection analysis of cis-genes and trans-genes revealed 1676 cis-specific genes, 441 trans-specific genes, and 422 shared genes (see Additional file 4: Figure S1a). Among the 422 shared genes, 378 had both significant cis- and trans-eQTL on the same chromosome (see Additional file 5: Table S4). Taking the *SLC5A4* gene as an example, we found that it was significantly associated with 28 cis-SNPs and with 400 trans-SNPs on *Sus scrofa* chromosome (SSC) SSC14 (see Additional file 4: Figure S1b). The top cis-SNP (SSC14:48,782,016, G > A) and the top trans-SNP (SSC14:45,347,247, A > G) displayed strong linkage disequilibrium with each other (D’ = 0.99 and r^2^ = 0.83), which suggests that the associations between these SNPs (both cis and trans SNPs) and the *SLC5A4* gene originated from a common cis-regulatory mutation. Among the 441 trans-specific genes, 399 genes had trans-eQTL on different chromosomes, e.g. *ENSSSCG00000035894* (see Additional file 4: Figure S1c), and 42 genes had trans-eQTL on the same chromosome, e.g. *STMN3* (*ENSSSCG00000038405*) (see Additional file 4: Figure S1d).

To evaluate the size of the cis-regulatory effect of each cis-eQTL with respect to the expression of the corresponding associated gene, for all characterized cis-eQTL, 38.04% (4068/10,693) had an absolute allele substitution effect estimate greater than 0.75, corresponding to approximately a 1.5-fold change in expression. For a subset of 1459 cis-eQTL, the absolute estimate of the allele substitution effect was greater than 1.0, corresponding to an approximately twofold change in expression (see Additional file 2: Table S2). The allele substitution effect estimates of all characterized cis-eQTL displayed an obviously distinct bimodal density distribution (Fig. 1c).

Estimates of the heritability for the expression level of each gene showed that cis-genes had significantly higher heritabilities (p < 2.2e-16, Student’s t test) (Fig. 1d) as genes without associated cis-eQTL (Fig. 1e), although expression levels were not significantly different between these two groups of genes (p = 0.072, Student’s t test).

An eQTL can influence the expression of multiple genes, which we refer to as pleiotropy of eQTL [40]. Descriptive statistics revealed that 29.77% (2,141/7,192) of cis-eQTL and 34.36% (2,199/6,400) of trans-eQTL were associated with the expression of two or more target genes, and 64 cis-eQTL and 107 trans-eQTL were associated with six or more genes (Fig. 1f). It appeared that the eQTL that displayed pleiotropy were distributed in some specific chromosome regions but formed clusters, which could be regarded as eQTL hotspots (Fig. 1g). The most striking cis-eQTL with pleiotropy was the T > G substitution on SSC12 at position 5,516,315 (accession ID in the dbSNP database: rs340671286), which was associated with nine genes on SSC12 (see Additional file 4: Figure S1e). The most notable trans-eQTL with pleiotropy was the A > G substitution on SSCX at position 22,972,736 (accession ID in the dbSNP database: rs332199038), which was associated with 56 genes (see Additional file 4: Figure S1f).

### ASE analysis based on RNAseq SNPs confirmed some of the cis-eQTL associated genes

**Fig. 2:**
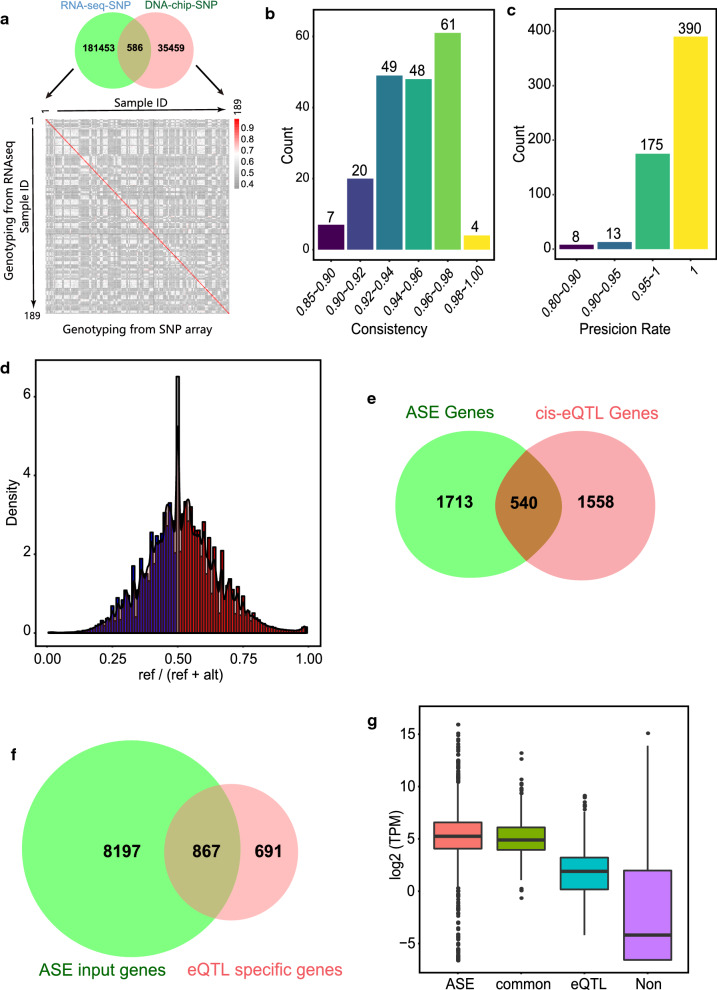
ASE analysis and overlap with cis-eQTL results. **a** Genotyping consistency between RNAseq SNPs and DNA chip SNPs. **b** Barplot of the genotyping consistency between RNAseq SNPs and DNA chip SNPs. **c** Barplot of the heterozygote genotyping precision rate of RNAseq SNPs. **d** Density distribution of the reference allelic ratio at each locus for all samples. A reference allelic ratio higher than 0.51 is shown in red, lower than 0.49 in blue and for other values in gray. **e** Venn diagram of cis-eQTL genes and ASE-eQTL genes. **f** Venn diagram of ASE input genes and cis-eQTL-specific genes. **g** Boxplot of gene expression levels of different classifications. “ASE” means all the ASE-specific genes, “eQTL” represents the cis-eQTL-specific genes, “Non” represents all genome genes that have neither associated eQTL nor ASE signals, and “common” represents all common genes that were identified by both eQTL and ASE analyses

To further confirm the results of the cis-eQTL, we identified all the SNPs in the RNA sequencing data (RNAseq SNPs). In total, we characterized 182,039 RNAseq-SNPs that passed QC, which included 586 SNPs that were also present on the Illumina porcine 50 K + SNP iSelect™ BeadChip (DNA-chip SNPs) (Fig. 2a). Consistency between the two genotyping methods (RNAseq versus DNA chip) using the common SNPs revealed that most individuals (182/189) had a consistency greater than 90%, and the remaining seven had a consistency greater than 85% (Fig. 2b). Furthermore, the RNAseq genotyping precision for heterozygotes was evaluated for 586 common SNPs, 578 of which had a precision rate higher than 90% (Fig. 2c). The overall RNAseq genotyping precision for all heterozygotes at the 586 common SNPs was 99.08%, which indicates a high reliability of RNAseq-based genotyping.

Prior to ASE analysis, we evaluated mapping bias by calculating the allelic ratio, which is the ratio between counts of reference allele specific reads and total counts of all reads; this displayed an approximately symmetrical distribution (Fig. 2d). We identified 9815 significant ASE SNPs in 2253 unique genes (see Additional file 6: Table S5), which represented 8.71% of all genes in the reference genome (25,880). Overlap analysis between ASE-associated genes and cis-eQTL genes identified 540 common genes (Fig. 2e), which means that 1558 cis-eQTL genes were not confirmed by ASE analysis, of which 691 genes displayed low expression levels and did not pass QC of ASE (Fig. 2f and g).

### Correlation analysis between gene expression and phenotypic value and reference analysis highlight candidate genes for meat-quality

To highlight potential candidate genes for meat quality traits, we analyzed the correlation between trait phenotypes and gene expression levels. Among the 13,450 genes, 768 genes had an expression level that was significantly correlated with at least one trait phenotype, including 232, 2, 12, 17, 118, and 387 genes that were correlated with average B value, average C value, average L value, average H value, pH at 45 min, and pH at 24 h, respectively (see Additional file 7: Table S6). Seventy-seven genes were correlated with more than one trait, e.g. *PYGM*, which encodes the muscle-associated glycogen phosphorylase, and the expression level of which was significantly correlated with pH 45 min (q = 6.92e-4) and average B value (q = 2.73e-2). Among the 768 genes that had an expression level correlated with trait phenotypes, 103 were cis-eQTL-associated genes and 138 were ASE genes (see Additional file 7: Table S6); 33 genes were identified by both cis-eQTL and ASE analyses (Table 1).

**Table 1:** The overlap between eQTL analysis, ASE analysis and correlation analysis

Gene_name	eQTL analysis		ASE analysis			Correlation analysis			
	SNP_id	Fdr	SNP_id	Num_Het	Num_ASE	Ratio	Traits	Cor	Fdr
*HUS1*	18:48910892	2.39E − 19	18:48526014	122	71	0.58	mean_B	−0.27	2.82E − 02
							mean_H	−0.30	4.03E − 02
*SPECC1*	12:59392739	3.75E − 14	12:59557437	120	26	0.22	pH_24h	0.25	3.18E − 02
							mean_B	−0.24	4.84E − 02
*METTL22*	3:34053010	1.99E − 11	3:34040204	78	25	0.32	mean_H	−0.29	4.49E − 02
*ZFAND2B*	15:120963216	4.85E − 06	15:121243305	74	73	0.99	pH_45min	−0.33	1.97E − 03
*PYGM*	2:7381573	1.19E − 04	2:7407552	109	102	0.94	pH_45min	0.35	6.92E − 04
							mean_B	0.28	2.73E − 02
*ENSSSCG00000009631*	14:7718361	4.29E − 04	14:6978183	160	50	0.47	pH_24h	−0.25	3.01E − 02
*ABRA*	4:30027414	4.36E − 04	4:30712640	108	107	0.99	mean_B	−0.28	2.59E − 02
*RSL1D1*	3:30749727	2.59E − 03	3:31285647	117	16	0.14	pH_24h	0.29	1.05E − 02
*CAV3*	13:64696229	3.04E − 03	13:65131086	122	97	0.80	pH_24h	−0.25	3.16E − 02
							mean_L	0.32	2.79E − 02
*GMPR*	7:12312194	3.07E − 03	7:12261459	126	124	0.98	pH_24h	−0.25	2.93E − 02
*NTN4*	5:86726806	3.83E − 03	5:87783446	89	11	0.12	mean_L	−0.36	6.25E − 03
*GPC1*	15:139006307	5.29E − 03	15:139483525	119	56	0.47	pH_45min	−0.26	4.99E − 02
*TRIM54*	3:111962587	7.51E − 03	3:111836687	108	36	0.33	mean_B	−0.26	4.08E − 02
							pH_24h	0.26	2.83E − 02
*LGMN*	7:114180118	8.75E − 03	7:114200066	145	40	0.28	pH_45min	−0.28	2.33E − 02
*CACNA1S*	10:23528382	1.14E − 02	10:23530021	133	71	0.53	pH_45min	0.26	4.43E − 02
*ENSSSCG00000020808*	3:16083309	1.59E − 02	3:16844732	109	41	0.38	mean_B	−0.27	2.73E − 02
*MTRR*	16:74265935	1.81E − 02	16:74245560	66	13	0.20	pH_24h	0.24	4.53E − 02
*CCT8*	13:192188992	1.83E − 02	13:192414026	58	12	0.21	mean_B	−0.27	2.96E − 02
*ZFAND2A*	3:930630	2.11E − 02	3:757286	106	21	0.20	pH_24h	0.25	3.18E − 02
*RRP7A*	5:6407132	2.17E − 02	5:6216045	137	44	0.32	mean_B	−0.28	2.73E − 02
*SOD2*	1:7722553	2.27E − 02	1:7679607	184	115	0.63	pH_24h	0.25	3.03E − 02
*ANKRD10*	11:78298873	2.37E − 02	11:77325740	94	10	0.11	pH_24h	0.24	4.35E − 02
*SDR39U1*	7:75705436	2.64E − 02	7:74851797	99	45	0.45	mean_B	0.24	4.84E − 02
*PLA2G7*	7:41182729	3.35E − 02	7:41498072	186	49	0.26	mean_B	−0.27	2.73E − 02
*NAT9*	12:5991040	3.53E − 02	12:6466570	131	53	0.40	mean_H	−0.30	4.03E − 02
*NPNT*	8:116112745	3.72E − 02	8:115860561	44	15	0.34	pH_24h	0.26	2.55E − 02
*GRK3*	14:44316076	3.76E − 02	14:43439845	68	33	0.49	pH_24h	0.25	3.33E − 02
*SMIM3*	2:150955856	3.95E − 02	2:151763489	121	11	0.09	mean_B	−0.25	4.48E − 02
*ACTN3*	2:6263381	4.35E − 02	2:5861691	117	109	0.93	pH_24h	−0.27	2.00E − 02
*NR4A1*	5:17110306	4.74E − 02	5:17405098	74	22	0.30	pH_45min	0.49	1.86E − 09
*CTGF*	1:31914264	4.75E − 02	1:31676377	96	18	0.19	pH_45min	0.27	2.77E − 02
*DYNLT1*	1:8669156	4.82E − 02	1:8562162	129	58	0.45	pH_24h	−0.30	8.57E − 03
*KLHL40*	13:27052606	4.90E − 02	13:26209704	86	33	0.38	pH_24h	0.30	9.62E − 03

**“**Num_Het” represents the total number of observed heterozygotes, and “Num_ASE” means the number of heterozygotes that displayed ASE

To check whether these cis-genes and ASE genes had ever been suggested as candidate genes for economic traits in the literature, we set “pig” and “GWAS” as the key words to search all publications in PubMed and collected 434 publications (until September 2019). Based on checking the abstract and main results, we selected 260 publications, from which we collected 1869 candidate genes. By overlap analysis, we confirmed that 201 cis-eQTL-associated genes (see Additional file 8: Table S7) and 250 ASE genes (see Additional file 9: Table S8) were considered as candidate genes of economic traits in previous studies, including 63 genes that were identified by both cis-eQTL and ASE analyses (see Additional file 10: Table S9).

## Discussion

We have conducted thorough analyses to understand the global genetic regulation of gene expression in porcine skeletal muscle via eQTL and ASE analyses. First, we applied a stringent QC procedure for both expression and genotyping data. The experimental population was the F1 population of two distant breeds, and the heterozygous genotype was the dominant genotype for some loci that were fixed for different alleles in two breeds. Thus, we did not perform a Hardy–Weinberg equilibrium (HWE) test for the genotyping data. Instead, for DNA-chip SNPs, we set a threshold of 19 for the median of the number of each genotype at each site to avoid an extreme distribution of genotypes. For ASE analysis with RNAseq data, we applied the N-masking alignment strategy to eliminate allelic mapping bias and set filtering conditions for both total read counts (10) and allele-specific read counts (3), as well as the minimum sample size of heterozygotes (30) and the count of ASE heterozygotes (10), which ensured higher reliability.

We found that 43.8% (378/863) of the trans-genes had a cis-eQTL on the same chromosome, such as *SLC5A4*. It should be noted that the distinction between cis- and trans-eQTL depends on the choice of the size of the cis-window [41], which was defined as 1 Mb on either side of the associated gene in our study. Therefore, we could not rule out the possibility that most (if not all) of the above-mentioned trans-eQTL affect the expression of target genes in cis. Our results confirmed the observation that cis-eQTL were easier to detect than trans-eQTL [41] and cis-eQTL were of main interest. Overlap analysis revealed that less than half of the cis-eQTL genes were confirmed by ASE analysis, which is in agreement with results from other studies [25, 42, 43]. The limited overlap between the two approaches could result from several factors. First, the markers involved differed, i.e. the GWAS used 36,045 SNPs from the DNA chip, of which most were in intergenic regions, whereas ASE analysis used 182,039 SNPs from the RNAseq data, of which most were within genes. As a result, the ASE analysis had a higher probability of discovering genes associated with regulatory variants than the eQTL analysis. Second, some of the lowly expressed genes were excluded from ASE analysis but kept for eQTL analysis (Fig. 2f and g). And third, both approaches may have been prone to detecting abnormal signals that were not caused by cis regulatory variants. For example, spurious cis-eQTL signals could result from copy number variations [44] or splicing mutations [45], while spurious ASE signals could result from imprinting [25] or from allelic mapping bias, although this was well controlled but not eliminated in our study (Fig. 2d).

The cis-eQTL analysis identified potential genes associated with unknown regulatory variations and ASE analysis can validate their cis-regulatory effect [25]. Therefore, in spite of the limited overlap between the identified cis-eQTL and ASE genes, combining these two approaches should have increased the reliability of the results. The eQTL approach considers the total gene expression as the phenotype, which is influenced not only by regulatory variations but also by trans-acting environmental and genetic factors [46]. The ASE approach uses heterozygotes to identify regulatory variants that alter allele-specific expression and contrasts the expression of two alleles that are in the same cellular environment and which can, therefore, be internal controls for each other, ensuring higher accuracy of the results [46]. As a result, ASE analysis can provide complementary and more precise mapping results than eQTL analysis, and combining these two approaches could speed up the identification of regulatory variants.

Gene expression level is a molecular phenotype that can bridge the gap between the gene and the tissue phenotype. In this study, we observed that the expression of some genes was correlated with various meat quality traits, which can be used in other studies, such as in prioritizing potential functional genes in GWAS. In particular, we highlighted 33 genes with expression levels that were correlated with meat quality traits, and for which their related eQTL were identified by both cis-eQTL and ASE analyses. These genes could be used as candidate genes for meat quality traits in future studies. In addition, we identified 63 candidate genes (see Additional file 10: Table S9) that were identified by the two approaches and that were reported in a previous publication. Among these 63 candidate genes, the most famous one is *PHKG1*, in which a splicing mutation (g.8283C > A) in Duroc pigs leads to increased glycolytic potential and rapid pH decline in meat [6]. In a previous study, we confirmed that the splicing mutation g.8283C > A is the causative mutation for the eQTL signal and that pigs that carry the mutant allele have a high risk of pale, soft, and exudative meat [30]. Other identified candidate genes that are of interest include *NUDT7*, which was assigned to a QTL for meat color [47–49], and *FADS2* and *DGAT2*, which are involved in the regulation of lipid metabolism and fat deposition [50–52]. The discovery of cis-eQTL that regulate the expression of these genes further confirms that these candidate genes affect economic traits in the pig and enhance possibilities to identify the causative mutations for each gene in future studies.

The animals used for muscle transcriptome sequencing were crossbred pigs between Duroc boars and Luchuan sows. An F1 population is generally considered not suitable for QTL mapping due to its limited genetic segregation and power. However, the Luchuan pig is a Chinese local pig breed that is not highly selected, and the use of a relatively large group of unrelated Luchuan sows (158 sows) represents extensive within-population diversity, which ensures that the regulatory effects of some genetic variations could be detected. Moreover, the ASE approach relies on heterozygous individuals, which are more abundant in an F1 population. In addition, gene expression is a molecular phenotype that has a higher detection sensitivity than classical trait phenotypes. Therefore, our results provide valuable information on the genetics of gene expression in skeletal muscle.

## Conclusions

We conducted intensive analyses of the global genetic regulation of gene expression in porcine skeletal muscle via eQTL and ASE analyses. By eQTL analysis, we identified 2098 cis-genes (p ≤ 1.33e-3, FDR ≤ 0.05) and 863 trans-genes (p ≤ 1.13e-6, FDR ≤ 0.05). ASE analysis based on RNAseq SNPs confirmed 540 cis-genes, which included 33 genes with expression levels that were correlated with at least one meat quality trait and 63 candidate genes that affect pig economic traits and that were identified in previous studies. The present study confirmed several previously published candidate genes and identified some novel candidate genes, which will advance the understanding of the genetics that underlie pig meat quality traits.

## Supplementary information

**Additional file 1: Table S1.** Library sizes and alignment information about 189 individuals.

**Additional file 2: Table S2.** Identified cis-eQTL.

**Additional file 3: Table S3.** Identified trans-eQTL.

**Additional file 4: Figure S1.** Supplementary results of genome-wide eQTL analysis. **a** Venn diagram of cis-eQTL genes and trans-eQTL genes. **b** Scatter plot of -log10(p values) of eQTL associated with SLC5A4 on SSC14. The red and blue dots represent cis-eQTL and trans-eQTL, respectively. Gray dotted lines indicate the cutoff value for cis-eQTL (p = 1.33e-3) and trans-eQTL (p = 1.13e-6). **c** Manhattan plot of *ENSSSCG00000035894.* The red dotted line represents the cutoff for trans-eQTL (p = 1.13e-6). The trans-eQTL SNPs located on SSC6 and their associated gene located on SSC9. **d** Manhattan plot of *STMN3.* Both the trans-eQTL SNPs and their associated genes were located on the same chromosome. **e** The circos plot of the cis-eQTL pleiotropic example. **f** The circos plot of the trans-eQTL pleiotropic example.

**Additional file 5: Table S4.** Common associated genes between cis-eQTL and trans-eQTL.

**Additional file** 6: **Table S5.** Results of the ASE-eQTL by ASE analysis.

**Additional file 7: Table S6.** Correlation analysis between seven meat quality traits and gene expression level.

**Additional file 8: Table S7.** Shared genes between cis-eQTL target genes and candidate genes.

**Additional file 9: Table S8.** Shared genes between ASE-eQTL target genes and candidate genes.

**Additional file 10: Table S9.** Overlap between eQTL analysis, ASE analysis and candidate genes.

## References

[1] Hindorff LA, Sethupathy P, Junkins HA, Ramos EM, Mehta JP, Collins FS (2009). Potential etiologic and functional implications of genome-wide association loci for human diseases and traits. Proc Natl Acad Sci USA.

[2] Hou L, Zhao H (2013). A review of post-gwas prioritization approaches. Front Genet.

[3] Vandiedonck C (2018). Genetic association of molecular traits: a help to identify causative variants in complex diseases. Clin Genet.

[4] Lawrenson K, Li Q, Kar S, Seo JH, Tyrer J, Spindler TJ (2015). Cis-eqtl analysis and functional validation of candidate susceptibility genes for high-grade serous ovarian cancer. Nat Commun.

[5] Skelly DA, Ronald J, Akey JM (2009). Inherited variation in gene expression. Annu Rev Genomics Hum Genet.

[6] Ma J, Yang J, Zhou L, Ren J, Liu X, Zhang H (2014). A splice mutation in the phkg1 gene causes high glycogen content and low meat quality in pig skeletal muscle. PLoS Genet.

[7] Karim L, Takeda H, Lin L, Druet T, Arias JA, Baurain D (2011). Variants modulating the expression of a chromosome domain encompassing plag1 influence bovine stature. Nat Genet.

[8] Brynedal B, Choi J, Raj T, Bjornson R, Stranger BE, Neale BM (2017). Large-scale trans-eqtls affect hundreds of transcripts and mediate patterns of transcriptional co-regulation. Am J Hum Genet.

[9] Christie N, Myburg AA, Joubert F, Murray SL, Carstens M, Lin YC (2017). Systems genetics reveals a transcriptional network associated with susceptibility in the maize-grey leaf spot pathosystem. Plant J.

[10] Kim YA, Przytycka TM (2012). Bridging the gap between genotype and phenotype via network approaches. Front Genet.

[11] Yin X, Cheng H, Lin Y, Fan X, Cui Y, Zhou F (2014). Five regulatory genes detected by matching signatures of eqtl and gwas in psoriasis. J Dermatol Sci.

[12] Nieuwenhuis MA, Siedlinski M, van den Berge M, Granell R, Li X, Niens M (2016). Combining genomewide association study and lung eqtl analysis provides evidence for novel genes associated with asthma. Allergy.

[13] Peters JE, Lyons PA, Lee JC, Richard AC, Fortune MD, Newcombe PJ (2016). Insight into genotype-phenotype associations through eqtl mapping in multiple cell types in health and immune-mediated disease. PLoS Genet.

[14] Joehanes R, Zhang X, Huan T, Yao C, Ying SX, Nguyen QT (2017). Integrated genome-wide analysis of expression quantitative trait loci aids interpretation of genomic association studies. Genome Biol.

[15] Pavlides JM, Zhu Z, Gratten J, McRae AF, Wray NR, Yang J (2016). Predicting gene targets from integrative analyses of summary data from GWAS and eQTL studies for 28 human complex traits. Genome Med.

[16] Guo H, Fortune MD, Burren OS, Schofield E, Todd JA, Wallace C (2015). Integration of disease association and eqtl data using a bayesian colocalisation approach highlights six candidate causal genes in immune-mediated diseases. Hum Mol Genet.

[17] Hormozdiari F, van de Bunt M, Segre AV, Li X, Joo JWJ, Bilow M (2016). Colocalization of gwas and eqtl signals detects target genes. Am J Hum Genet.

[18] Zhu Z, Zhang F, Hu H, Bakshi A, Robinson MR, Powell JE (2016). Integration of summary data from gwas and eqtl studies predicts complex trait gene targets. Nat Genet.

[19] Wimmers K, Murani E, Ponsuksili S (2010). Functional genomics and genetical genomics approaches towards elucidating networks of genes affecting meat performance in pigs. Brief Funct Genomics.

[20] Ponsuksili S, Murani E, Schwerin M, Schellander K, Wimmers K. Identification of expression qtl (eqtl) of genes expressed in porcine m. *longissimus dorsi* and associated with meat quality traits. BMC Genomics. 2010;11:572. 10.1186/1471-2164-11-572 PMC3091721 20950486

[21] Liaubet L, Lobjois V, Faraut T, Tircazes A, Benne F, Iannuccelli N (2011). Genetic variability of transcript abundance in pig peri-mortem skeletal muscle: eQTL localized genes involved in stress response, cell death, muscle disorders and metabolism. BMC Genomics.

[22] Steibel JP, Bates RO, Rosa GJ, Tempelman RJ, Rilington VD, Ragavendran A (2011). Genome-wide linkage analysis of global gene expression in loin muscle tissue identifies candidate genes in pigs. PLoS ONE.

[23] Martinez-Montes AM, Muinos-Buhl A, Fernandez A, Folch JM, Ibanez-Escriche N, Fernandez AI (2017). Deciphering the regulation of porcine genes influencing growth, fatness and yield-related traits through genetical genomics. Mamm Genome.

[24] Kommadath A, Bao H, Choi I, Reecy JM, Koltes JE, Fritz-Waters E (2017). Genetic architecture of gene expression underlying variation in host response to porcine reproductive and respiratory syndrome virus infection. Sci Rep.

[25] Maroilley T, Lemonnier G, Lecardonnel J, Esquerre D, Ramayo-Caldas Y, Mercat MJ (2017). Deciphering the genetic regulation of peripheral blood transcriptome in pigs through expression genome-wide association study and allele-specific expression analysis. BMC Genomics.

[26] Velez-Irizarry D, Casiro S, Daza KR, Bates RO, Raney NE, Steibel JP (2019). Genetic control of longissimus dorsi muscle gene expression variation and joint analysis with phenotypic quantitative trait loci in pigs. BMC Genomics.

[27] Drag MH, Kogelman LJ, Maribo H, Meinert L, Thomsen PD, Kadarmideen HN (2019). Characterisation of eqtls associated with androstenone by rna sequencing in porcine testis. Physiol Genomics.

[28] Hu YJ, Sun W, Tzeng JY, Perou CM (2015). Proper use of allele-specific expression improves statistical power for cis-eqtl mapping with rna-seq data. J Am Stat Assoc.

[29] Khansefid M, Pryce JE, Bolormaa S, Chen Y, Millen CA, Chamberlain AJ (2018). Comparing allele specific expression and local expression quantitative trait loci and the influence of gene expression on complex trait variation in cattle. BMC Genomics.

[30] Liu Y, Liu Y, Ma T, Long H, Niu L, Zhang X (2019). A splicing mutation in *PHKG1* decreased its expression in skeletal muscle and caused PSE meat in Duroc x Luchuan crossbred pigs. Anim Genet.

[31] Purcell S, Neale B, Todd-Brown K, Thomas L, Ferreira MA, Bender D (2007). Plink: a tool set for whole-genome association and population-based linkage analyses. Am J Hum Genet.

[32] Browning BL, Zhou Y, Browning SR (2018). A one-penny imputed genome from next-generation reference panels. Am J Hum Genet.

[33] Kim D, Langmead B, Salzberg SL (2015). Hisat: a fast spliced aligner with low memory requirements. Nat Methods.

[34] Pertea M, Pertea GM, Antonescu CM, Chang TC, Mendell JT, Salzberg SL (2015). Stringtie enables improved reconstruction of a transcriptome from rna-seq reads. Nat Biotechnol.

[35] Shabalin AA (2012). Matrix eqtl: ultra fast eqtl analysis via large matrix operations. Bioinformatics.

[36] Quinlan AR, Hall IM (2010). Bedtools: a flexible suite of utilities for comparing genomic features. Bioinformatics.

[37] Castel SE, Levy-Moonshine A, Mohammadi P, Banks E, Lappalainen T (2015). Tools and best practices for data processing in allelic expression analysis. Genome Biol.

[38] Salavati M, Bush SJ, Palma-Vera S, McCulloch MEB, Hume DA, Clark EL (2019). Elimination of reference mapping bias reveals robust immune related allele-specific expression in crossbred sheep. Front Genet.

[39] Cingolani P, Platts A, Le Wang L, Coon M, Nguyen T, Wang L (2012). A program for annotating and predicting the effects of single nucleotide polymorphisms, snpeff: snps in the genome of drosophila melanogaster strain w1118; iso-2; iso-3. Fly.

[40] Tian J, Keller MP, Broman AT, Kendziorski C, Yandell BS, Attie AD (2016). The dissection of expression quantitative trait locus hotspots. Genetics.

[41] Ponsuksili S, Murani E, Phatsara C, Schwerin M, Schellander K, Wimmers K (2010). Expression quantitative trait loci analysis of genes in porcine muscle by quantitative real-time RT-PCR compared to microarray data. Heredity.

[42] Hasin-Brumshtein Y, Hormozdiari F, Martin L, van Nas A, Eskin E, Lusis AJ (2014). Allele-specific expression and eqtl analysis in mouse adipose tissue. BMC Genomics.

[43] Lagarrigue S, Martin L, Hormozdiari F, Roux PF, Pan C, van Nas A (2013). Analysis of allele-specific expression in mouse liver by rna-seq: a comparison with cis-eqtl identified using genetic linkage. Genetics.

[44] DeBoever C, Li H, Jakubosky D, Benaglio P, Reyna J, Olson KM (2017). Large-scale profiling reveals the influence of genetic variation on gene expression in human induced pluripotent stem cells. Cell Stem Cell.

[45] Lalonde E, Ha KC, Wang Z, Bemmo A, Kleinman CL, Kwan T (2011). RNA sequencing reveals the role of splicing polymorphisms in regulating human gene expression. Genome Res.

[46] Kang EY, Martin LJ, Mangul S, Isvilanonda W, Zou J, Ben-David E (2016). Discovering single nucleotide polymorphisms regulating human gene expression using allele specific expression from RNA-seq data. Genetics.

[47] Taniguchi M, Hayashi T, Nii M, Yamaguchi T, Fujishima-Kanaya N, Awata T (2010). Fine mapping of quantitative trait loci for meat color on sus scrofa chromosome 6: analysis of the swine *NUDT7* gene. J Anim Sci.

[48] Taniguchi M, Hayashi T, Nii M, Yamaguchi T, Fujishima-Kanaya N, Awata T (2010). Overexpression of *NUDT7*, a candidate quantitative trait locus for pork color, downregulates heme biosynthesis in l6 myoblasts. Meat Sci.

[49] Liu X, Xiong X, Yang J, Zhou L, Yang B, Ai H (2015). Genome-wide association analyses for meat quality traits in chinese erhualian pigs and a western duroc x (Landrace x Yorkshire) commercial population. Genet Sel Evol.

[50] Zhang J, Zhang Y, Gong H, Cui L, Huang T, Ai H, et al. Genetic mapping using 1.4 M SNP array refined loci for fatty acid composition traits in chinese erhualian and bamaxiang pigs. J Anim Breed Genet. 2017;134:472-83. 10.1111/jbg.12297 28940847

[51] Zhang W, Bin Y, Zhang J, Cui L, Ma J, Chen C (2016). Genome-wide association studies for fatty acid metabolic traits in five divergent pig populations. Sci Rep.

[52] Ma Y, Zhang S, Zhang K, Fang C, Xie S, Du X, et al. Genomic analysis to identify signatures of artificial selection and loci associated with important economic traits in duroc pigs. G3 (Bethesda). 2018. 8:3617-25. 10.1534/g3.118.200665 PMC6222590 30237295

