# Supplementary figures and images for: Genome-wide analysis of expression QTL (eQTL) and allele-specific expression (ASE) in pig muscle identifies candidate genes for meat quality traits
Source: Genet Sel Evol. 2020 Oct 9;52:59. doi: 10.1186/s12711-020-00579-x (PMC7547458; doi:10.1186/s12711-020-00579-x)

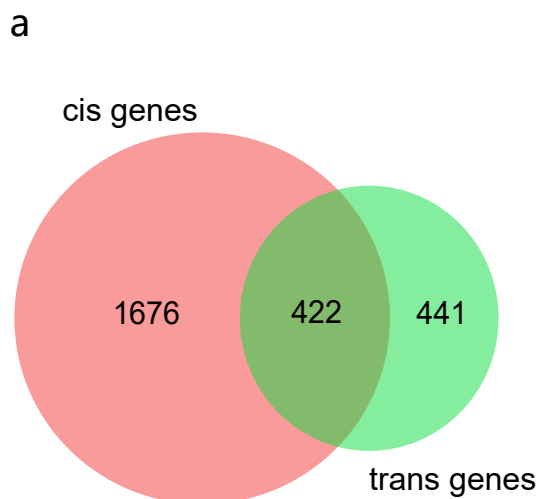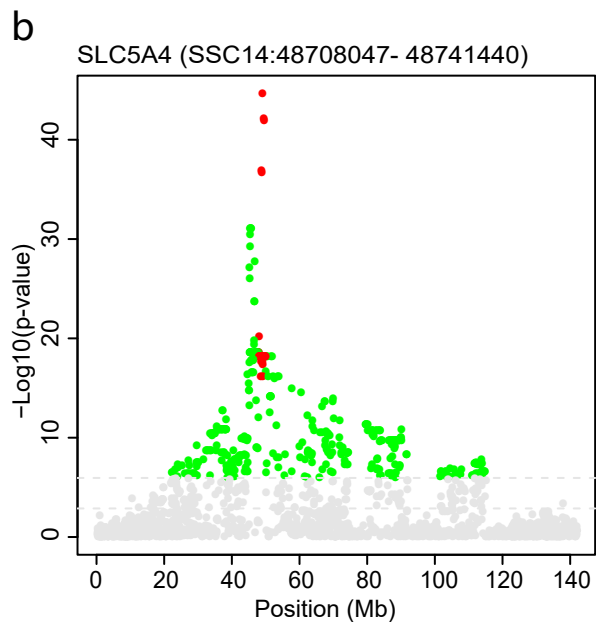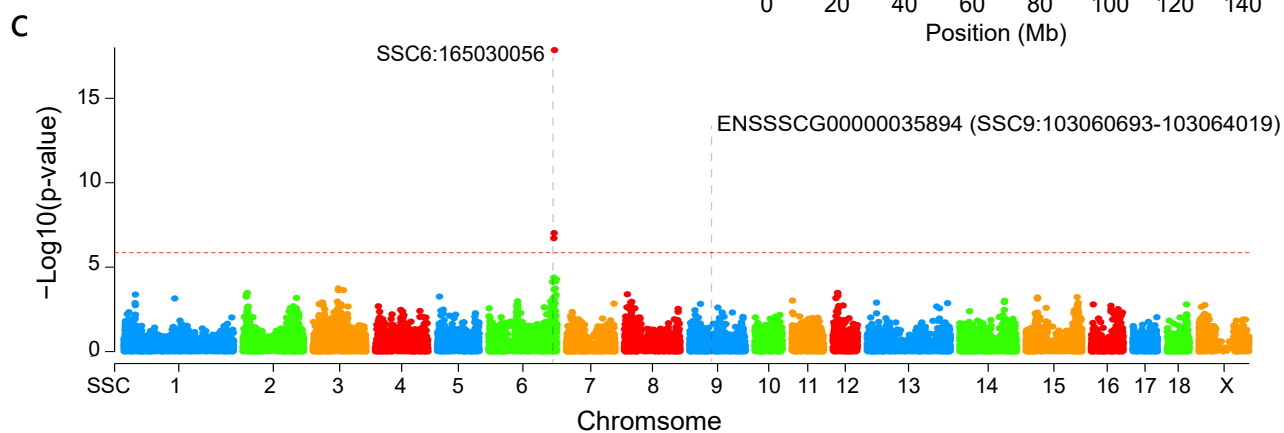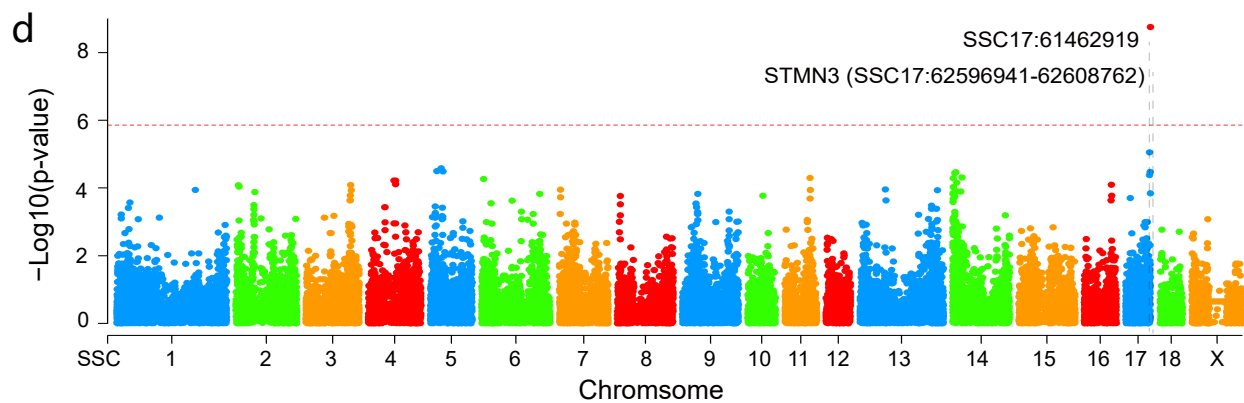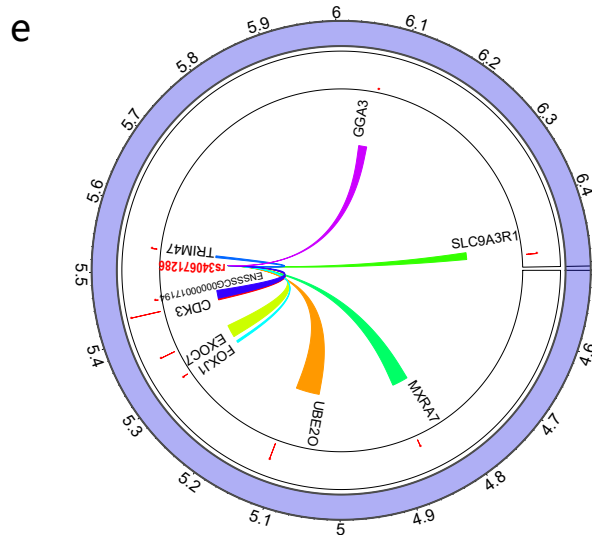

Supplement: Supplementary file 4 — Additional file 4: Figure S1. Supplementary results of genome-wide eQTL analysis. a Venn diagram of cis-eQTL genes and trans-eQTL genes. b Scatter plot of -log10(p values) of eQTL associated with SLC5A4 on SSC14. The red and blue dots represent cis-eQTL and trans-eQTL, respectively. Gray dotted lines indicate the cutoff value for cis-eQTL (p = 1.33e-3) and trans-eQTL (p = 1.13e-6). c Manhattan plot of ENSSSCG00000035894. The red dotted line represents the cutoff for trans-eQTL (p = 1.13e-6). The trans-eQTL SNPs located on SSC6 and their associated gene located on SSC9. d Manhattan plot of STMN3. Both the trans-eQTL SNPs and their associated genes were located on the same chromosome. e The circos plot of the cis-eQTL pleiotropic example. f The circos plot of the trans-eQTL pleiotropic example. [file 12711_2020_579_MOESM4_ESM.pdf]
